# Dynamic cfDNA Analysis by NGS in *EGFR* T790M-Positive Advanced NSCLC Patients Failed to the First-Generation EGFR-TKIs

**DOI:** 10.3389/fonc.2021.643199

**Published:** 2021-03-25

**Authors:** Li Ma, Haoyang Li, Dongpo Wang, Ying Hu, Mengjun Yu, Quan Zhang, Na Qin, Xinyong Zhang, Xi Li, Hui Zhang, Yuhua Wu, Jialin Lv, Xinjie Yang, Ruoying Yu, Shucai Zhang, Jinghui Wang

**Affiliations:** ^1^ Department of Medical Oncology, Beijing Tuberculosis and Thoracic Tumor Research Institute, Beijing Chest Hospital, Capital Medical University, Beijing, China; ^2^ Department of Radiology, Beijing Tuberculosis and Thoracic Tumor Research Institute, Beijing Chest Hospital, Capital Medical University, Beijing, China; ^3^ Research and Development, Nanjing Geneseeq Technology Inc., Nanjing, China; ^4^ Cancer Research Center, Beijing Tuberculosis and Thoracic Tumor Research Institute, Beijing Chest Hospital, Capital Medical University, Beijing, China

**Keywords:** circulating cell-free DNA (cfDNA), *EGFR*, T790M, third-generation EGFR TKI declarations, lung cancer

## Abstract

**Purpose:**

Circulating cell-free DNA (cfDNA) level has been demonstrated to be associated with efficacy in first generation EGFR TKIs in non-small cell lung cancer (NSCLC). However, the role of dynamic cfDNA analysis using next-generation sequencing (NGS) in patients with subsequent third-generation EGFR TKIs remains unclear.

**Methods:**

From 2016 to 2019, 81 NSCLC patients with *EGFR* T790M mutation either in tissue or plasma who received third-generation EGFR TKIs treatment were enrolled. CfDNA were sequenced by NGS with a 425-gene panel. The association of clinical characteristics, pretreatment, dynamic cfDNA and T790M level with outcomes in patients treated with the third-generation TKIs were analyzed.

**Results:**

In univariate analysis, the median PFS of patients with undetectable cfDNA level during treatment was significantly longer than those with detectable cfDNA (16.97 *vs*. 6.10 months; HR 0.2109; *P* < 0.0001). The median PFS of patients with undetectable T790M level during treatment was significantly longer than those with detectable T790M (14.1 *vs*. 4.4 months; HR 0.2192; *P* < 0.001). Cox hazard proportion model showed that cfDNA clearance was an independent predictor for longer PFS (HR 0.3085; *P* < 0.001) and longer OS (HR 0.499; *P* = 0.034). The most common resistant mutations of the third-generation TKIs were *EGFR* C797S (24%). *CDK6* CNV, *GRIN2A*, *BRCA2, EGFR* D761N, *EGFR* Q791H, *EGFR* V843I, and *ERBB4* mutation genes may possibly be new resistant mechanisms.

**Conclusions:**

Patients with undetectable cfDNA during the third-generation EGFR TKI treatment have superior clinical outcomes, and dynamic cfDNA analysis by NGS is valuable to explore potential resistant mechanisms.

## Introduction

*EGFR* gene mutations account for the most frequency of oncogenic mutations in advanced non-small cell lung cancer (NSCLC). The first-generation EGFR TKIs, gefitinib, erlotinib, icotinib, have been the standard-of-care as the first-line treatment in China for advanced NSCLC with *EGFR-*sensitizing mutations. Unfortunately, acquired resistance still occurs in the majority of patients. About 50–60% resistant mechanism of the first generation EGFR TKIs was *EGFR* T790M gene mutation. Third-generation EGFR TKIs, such as osimertinib, alflutinib, target the T790M mutation with outstanding clinical efficacy (1, 2).

As a viable alternative for tissue biopsy, liquid biopsy with circulating cell-free DNA (cfDNA) analysis has been used in identifying molecular target, predicting response and prognosis, and monitoring resistance during targeted treatment for lung cancer (3). cfDNA clearance during anticancer therapies has been an emerging topic in lung cancer, especially in EGFR-mutated NSCLCs. Previous studies have reported a promising role of cfDNA longitudinal monitoring using different liquid biopsy methodologies (4–7). The longitudinal cfDNA monitoring has been proposed as a follow-up tool in the identification of acquired mechanisms of resistance that can impact sequential treatment management (5, 8). Furthermore, several studies have reported cfDNA clearance as a potential predictive biomarker for NSCLC patients during immunotherapy (9).

The role of cfDNA dynamic changing in predicting the response to the third-generation EGFR TKIs has been investigated in small-scale studies with limited sample size using PCR technique (10). Due to the limitation of obtaining tissue samples at disease progression (PD), ctDNA pretreatment and at PD time point provide hints for better understanding the EGFR TKI resistant mechanisms (11, 12). Compared to conventional PCR-based testing, next-generation sequencing (NGS) is a more sensitive and comprehensive technique for detecting uncommon actionable mutations including acquired resistance alterations. Currently, there is a lack of research on dynamic detection of cfDNA by NGS in patients treated with the third-generation EGFR TKI, which limited the exploration of resistant mechanisms and the formulation of subsequent treatment regimens (13).

In this study, 81 *EGFR*-mutant advanced NSCLC patients who were confirmed to have acquired T790M mutation were enrolled, and the clinical implications of dynamic plasma cfDNA analysis using a 425-gene capture panel during the third-generation TKI treatment was investigated. The results implied a broad potential of cfDNA as an adjuvant tool in practical clinical management, regarding both prognosis prediction and resistant mechanism identification.

## Methods

### Patient Cohort

Advanced NSCLC patients with *EGFR* T790M identified in the tissue or plasma by NGS and prior-first-generation EGFR TKI treatment were enrolled. All patients received the third-generation TKIs sequentially. Key exclusion criteria were cases without NGS testing for tissue or plasma, or without disease evaluation. The study was approved by the ethics committee of Beijing Chest Hospital, and all patients had signed written informed consent. Clinical staging complied with the AJCC 8^th^ staging system, and treatment response was assessed with the Response Evaluation Criteria in Solid Tumors (RECIST), version 1.1. Blood samples were collected at the point of pretreatment and during the treatment of the third-generation EGFR TKIs.

### DNA Extraction and Targeted Next-Generation Sequencing

Genomic DNA from FFPE samples and white blood cells were extracted using the QIAamp DNA FFPE Tissue Kit (Qiagen). Plasma was extracted from about 10 ml whole blood in EDTA-coated tubes within 2 h of blood withdrawing, and circulating cell free DNA (cfDNA) was extracted using the QIAamp Circulating Nucleic Acid Kit (QIAGEN). Genomic DNA from white blood cells was extracted using DNeasy Blood & Tissue Kit (Qiagen, Germany) and used as normal control. All DNA concentration and purity were qualified by Nanodrop2000 (Thermo Fisher Scientific). All DNA samples were also quantified by Qubit 3.0 using the dsDNA HS Assay Kit (Life Technologies) according to the manufacturer’s protocol. The median yield for the plasma cfDNA at baseline was 15.68 ng/ml and 18.05ng/ml at disease progression.

Sequencing libraries were constructed using KAPA Hyper Prep kit (KAPA Biosystems) with an optimized manufacturer’s instructions. In brief, cfDNA or DNA was experienced with end-repairing, A-tailing, adapter ligation and size selection using Agencourt AMPure XP beads (Beckman Coulter). Libraries were then subjected to PCR amplification and purification before targeted enrichment. The size distribution of libraries was measured by Agilent Technologies 2100 Bioanalyzer (Agilent Technologies). The enriched libraries were sequenced on Illumina Hiseq 4000 NGS platforms to cover mean depths of at least 1,000×, 3,000×, and 100×, for FFPE, cfDNA, and blood, respectively. DNA extraction and NGS were performed in Geneseeq Technology Inc. A complete list of the genes included in the NGS panel was in **Supplementary Table 1**.

### Data Processing

Trimmomatic was used for FASTQ file quality control (QC). Leading/trailing low quality (quality reading below 30) or N bases were removed. Remaining reads were mapped to the reference sequence data (Human Genome version 19) using Burrows-Wheeler Aligner (BWA-mem, v0.7.12). Indel realignment and base quality score recalibration were performed with Genome Analysis Toolkit (GATK 3.4.0). Somatic mutations were detected with VarScan2. Copy number variations (CNVs) were detected using ADTEx (http://adtex.sourceforge.net) with default parameters as reported by previous studies (14–16). To eliminate sequencing artifact, a local bioinformatics pipeline was performed. Firstly, we used a local white blood cell (WBCs) database, containing recurrent somatic alterations from WBCs of 400 patients, to eliminate the sequencing artifacts. Specifically, if a variant was detected (*i.e.* ≥2 mutant reads) in >10% of the samples, it was considered a likely artifact and was removed. Secondly, a background denoising strategy was performed. Briefly, we performed panel sequencing with similar sequencing depth used in this study on plasma samples of 50 healthy individuals to assemble a database of alterations at each site of the panel and build a background error model. A specific alteration at a specific site was considered sequencing noise if the allele frequency (AF) and distinct supporting reads were not significantly beyond the background error probability. Only alteration with an AF over three standard deviations from the mean AF of healthy plasma-cfDNAs pool remained and subjected for further analyze.

### Bioinformatics Analyses

Trimmomatic48 was used for FASTQ file quality control (QC) (17). Sequencing data were aligned to the reference hg19 (Human Genome version 19) with the Burrows-Wheeler Aligner (BWA-mem, v0.7.12) (18). SNVs and Indels were detected using SCALPEL (http://scalpel.sourceforge.net) and Genome Analysis Toolkit (GATK). Tumor purity was estimated by ABSOLUTE (19). Purity-adjusted gene-level and segment-level copy numbers were calculated by CNVKit (20) ADTEx (http://adtex.sourceforge.net).

### Statistical Analysis

Progression-free survival (PFS) was defined as the first date of third-generation EGFR TKIs until disease progression or death resulting from any cause. Overall survival (OS) was defined as the first date of third-generation EGFR TKIs until death from any cause. CfDNA level was defined as the sum of total cfDNA total mutation abundance detected by the 425-gene panel NGS. Similarly, T790M level was reflecting the mutation abundance detected by the 425-gene panel. Comparisons were made using an unpaired two-tailed t-test, and analysis of variance (one-way ANOVA) was performed using Graphpad Prism V8.0, and linear regression analysis and some survival analysis were also analyzed using Prism V8.0. Most of the statistical analyses were performed using R version 3.5.3. In particular, Survival, Survminer, and Cox analysis were used for the analysis of PFS and OS. All reported *P* values were two-tailed, and *P* values less than 0.05 were considered statistically significant.

## Results

### Patient Characteristics and Samples

**Figure 1:**
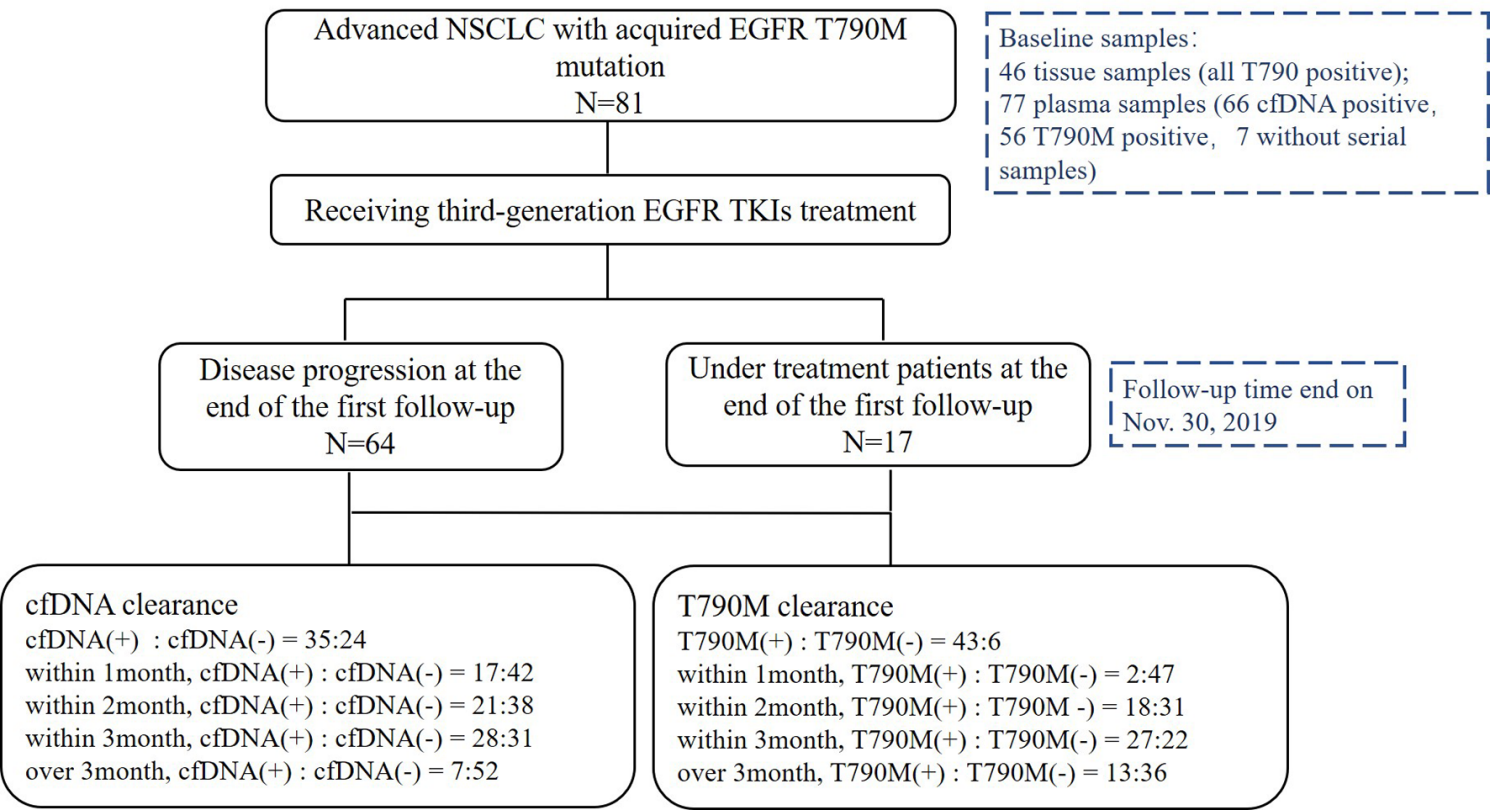
Patient enrollment.

**Figure 2:**
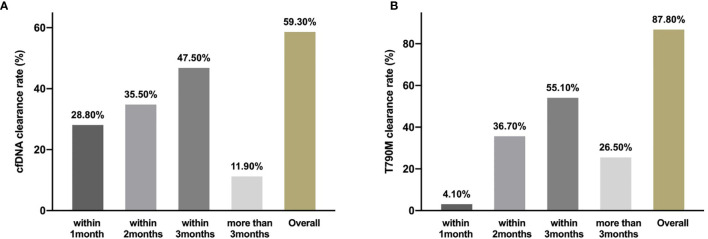
Clearing rate of cfDNA and *EGFR* T790M. The clearance rate of cfDNA **(A)** and EGFR T790M **(B)** within 1month, months and more than 3months during the third generation TKIs treatment.

From May 2016 to November 2019, 81 patients with a median age of 64 years old (range, 36–84) were enrolled. Twenty-six patients (32.1%) were male, 79 (97.5%) were adenocarcinoma, 16 (19.8%) were smokers, 77 (95.1%) were stage IV, 27 (33.3%) patients had central nervous system metastasis, and 57 (70.4%) were in second-line setting (**Table 1**). Above all, 64 patients (79.0%) were disease progression and 33 patients (40.7%) died at the last follow-up time on Nov. 30, 2019. Baseline samples were taken before the third-generation TKI treatment. Baseline tissue samples were obtained in 46 patients and identified with *EGFR* T790M. Baseline plasma samples were obtained in 77 patients. Sixty-six patients were cfDNA positive at baseline including 56 T790-positive cases. Fifty-nine patients had serial plasma samples available (**Figure 1**). Forty-one patients received osimertinib (80 mg, qd) and 40 patients received alflutinib (80 mg, qd). Clearing rates of cfDNA and *EGFR* T790M over the third-generation TKI treatment course were shown in **Figure 2**. The clearance of cfDNA as well as *EGFR*-T790M increased from the treatment onset and reached the maximum at three months, suggesting these patients responded to the treatment.

**Table 1:** Patient characteristics at pretreatment of third-generation EGFR TKIs.

Variables	N	%
Sex		
Male	26	32.1
Female	55	67.9
Age		
Median (range)	64 (36–84)	
≤60	32	39.5
>60	49	60.5
Histology		
Adenocarcinoma	79	97.5
Squamous adenocarcinoma	1	1.2
Non-small cell lung cancer NOS	1	1.2
Smoking status		
Non-smoking	65	80.2
Smoking	16	19.8
Clinical Staging		
IIIB	4	4.9
IV	77	95.1
Central and nervous system metastasis		
Yes	27	33.3
No	54	66.7
Treatment line		
Second line	57	70.4
Third line	15	18.5
Beyond third line	9	11.1

### Predictors of Progression-Free Survival

The median PFS of 81 patients was 14.167 months (95%CI 11.704–16.630). There was no significant correlation between sex, age, smoking history, clinical staging, CNS metastasis, or treatment-line with the PFS of the third-generation TKIs, respectively (**Supplementary Table 2**). Histology type was not analyzed because 79 of 81 patients were adenocarcinoma.

**Figure 3:**
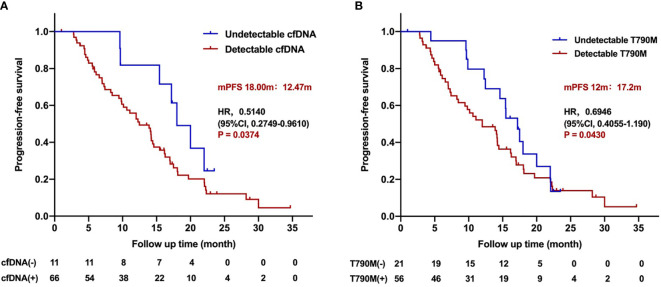
Progression-free survival for patients treated with the third-generation EGFR TKIs according to pretreatment cfDNA and T790M level. **(A)** Kaplan–Meier analysis stratified by cfDNA level. **(B)** Kaplan–Meier analysis stratified by T790M level.

Interestingly, univariate analysis showed that the median PFS of the patients with pretreatment undetectable cfDNA was significantly longer than those with detectable cfDNA (18.0 *vs*. 12.47 months; HR 0.5140, 95%CI, 0.2749–0.9610; *P* = 0.0374; **Figure 3A**). The PFS of patients with pretreatment undetectable T790M was superior to those with detectable T790M patients (17.2 *vs*. 12 months; HR 0.6946, 95%CI 0.4055–1.190; *P* = 0.0430; **Figure 3B**). However, multivariate analysis showed that neither pretreatment cfDNA level (HR 0.7759, 95%CI 0.3275–1.828; *P* = 0.564) nor pretreatment T790M level (HR 1.0535, 95%CI 0.4927–2.253; *P* = 0.893) was associated with the PFS for patients who received the third-generation EGFR TKIs treatment.

**Figure 4:**
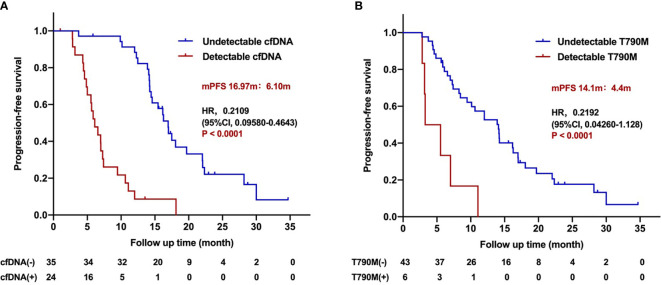
Progression-free survival for patients treated with the third-generation EGFR TKIs according to dynamic cfDNA and T790M level during treatment. **(A)** Kaplan–Meier analysis stratified by cfDNA level. **(B)** Kaplan–Meier analysis stratified by T790M level.

Further analysis was carried out to study the association of dynamic changing of cfDNA with PFS. In 59 cfDNA-positive patients who had serial plasma samples, 35 patients presented with cfDNA clearance. The clearance rate of cfDNA level within 1, 2, 3, and beyond 3 months during the third-generation TKI treatment was 28.8% (17/59), 35.5% (21/59), 47.5% (28/59), and 11.9% (7/59), respectively. The PFS of patients with undetectable fDNA during treatment was significantly longer than that of patients with detectable cfDNA (16.97 *vs*. 6.10 months; HR 0.2109, 95%CI 0.09580–0.4643; *P* < 0.0001; **Figure 4A**). The PFS of patients with undetectable cfDNA during the entire time of treatment was significantly longer than that with detectable cfDNA within post-treatment of 1, 2, 3, or more than 3 months, respectively (**Supplementary Figure 1**). Nevertheless, there were no significant differences on PFS among patients with undetectable cfDNA in 1, 2, 3, or more than 3 months after the third-generation TKI treatment. The duration of undetectable cfDNA was also positively correlated with the longer PFS during the third-generation TKI treatment (*R* ^2^ = 0.4428; *P* < 0.0001; **Supplementary Figure 2**).

In 49 T790M-positive patients who had serial plasma samples, 43 patients developed T790M clearance. Patients with undetectable T790M during treatment benefited from the third-generation TKI treatment compared with those with detectable T790M patients (14.1 *vs*. 4.4 months; HR 0.2192, 95%CI 0.04260–1.128; *P* < 0.001; **Figure 4B**). The relationship between T790M clearance speed and PFS is classified within 1, 2, 3 months, and more than 3 months after the third-generation TKI treatment (**Supplementary Figure 3**). The clearance rate of T790M level within 1, 2, 3 and beyond 3 months during the third-generation TKI treatment was 4.1% (2/49), 36.7% (18/49), 55.1% (27/49), and 26.5% (13/49). Among them, one patient did not receive continuous dynamic monitoring, and clearance was almost 1 year after treatment, which was included in the total number of clearance, but not in the calculation of different time points. The PFS stratified by T790M level at different times of treatment was shown in (**Supplementary Figure 4**). The duration of undetectable T790M was also positively correlated with longer PFS of the third-generation TKIs (R^2^ = 0.7235; *P* < 0.0001; **Supplementary Figure 4**).

In multivariate analysis, undetectable cfDNA level or T790M level during treatment was performed by Cox analysis. The results showed that undetectable cfDNA level during treatment was an independent predictor for longer PFS of the third-generation TKIs (HR 0.3085, 95%CI 0.1617–0.5885; *P* = 0.000359), while undetectable T790M was not (HR 0.57, 95%CI 0.2874–1.1303; *P* = 0.1075). These results suggest that dynamic cfDNA level can predict the efficacy of the third-generation TKIs, and negative cfDNA during treatment is associated with better outcome.

### Predictors of Overall Survival

**Figure 5:**
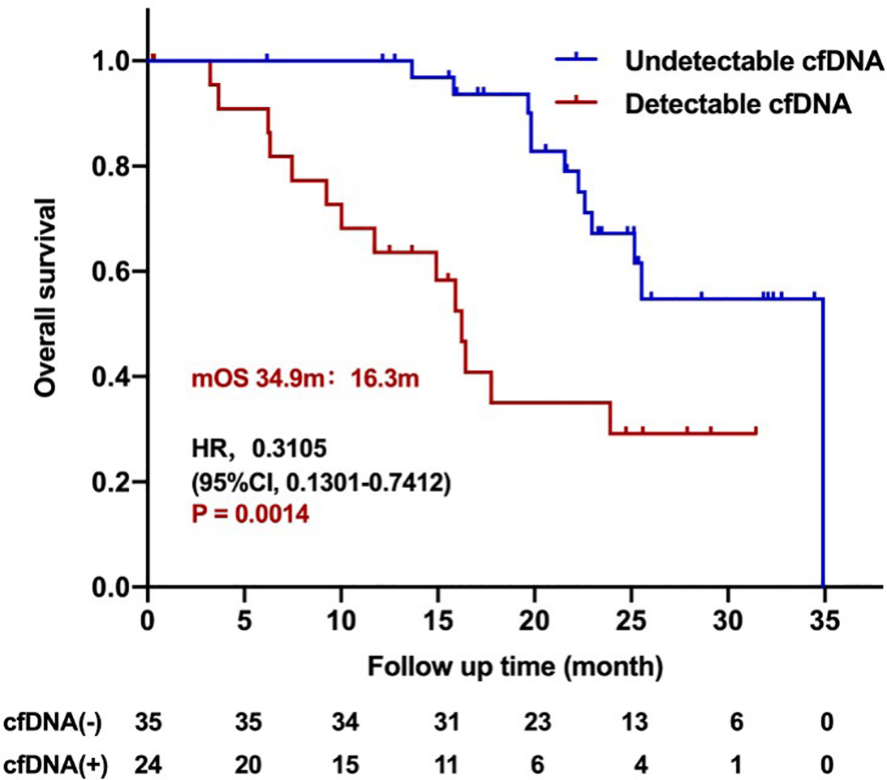
Kaplan–Meier analysis of overall survival for patients treated with the third-generation EGFR TKIs according to dynamic cfDNA level during treatment.

The median overall survival was 25.533 months (95%CI 22.615–28.452). Unfortunately, there were no significant correlations between clinical characteristics and over survival time (**Supplementary Table 2**), and no association was observed between pretreatment cfDNA level (HR 0.3525, 95%CI 0.1361–0.9129; *P* = 0.1336) or T790M level (HR 0.3680, 95%CI 0.1690–0.8012; *P* = 0.0498) and overall survival time. The cfDNA clearance was associated with the OS (34.9 *vs*. 16.3 months; HR 0.3105, 95%CI 0.1301–0.7412; *P* = 0.0014; **Figure 5**). Dynamic T790M level changes were not correlated with the OS (HR 0.6472, 95%CI 0.1127–3.716; *P* = 0.5509). These results demonstrated that detectable cfDNA during treatment is a poor factor of overall survival.

### Relationship of Cell-Free DNA Changing With Imaging (Chest CT)

The association of dynamic cfDNA changes with medical imaging presentation of patients who developed resistance to the third-generation TKIs was analyzed. The result showed that re-detected or re-elevated cfDNA level occurred earlier than progression on clinical imaging in 33 patients with 4.8 months in advance (*P* < 0.0001; **Supplementary Figure 5**), which may predict disease progression and recurrence risk.

### Resistance Mechanisms Detected by Next-Generation Sequencing

**Figure 6:**
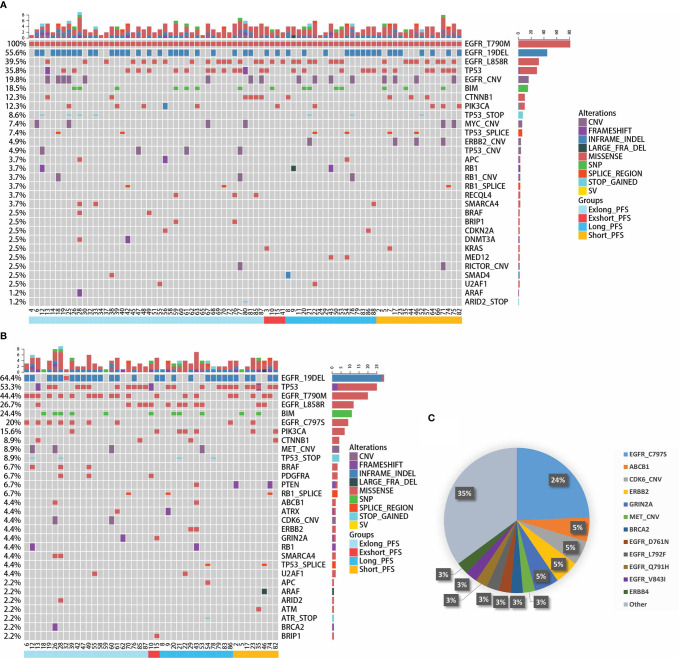
Gene mutation profile based on next-generation sequencing. **(A)** Somatic gene mutations profile containing the top 30 genes at pretreatment of the third-generation EGFR TKIs. **(B)** Somatic gene mutations profile containing the top 30 genes at disease progression point for patients with third-generation EGFR TKIs treatment. **(C)** Resistant gene mutation profile of third-generation TKIs.

In this cohort, thirty-nine patients had matched plasma and tumor tissue at baseline. Overall concordance, defined as the proportion of patients for whom at least one identical genomic alteration was identified in both tissue and plasma (21), was 69.2%(27/39). For *EGFR* T790M, 53.8% (21/39) of patients with matched plasma and tissue had T790M identified in both samples. The gene mutation profiles at baseline and PD point during the third-generation TKI treatment were further explored. The top 30 somatic mutational genes at pretreatment were showed in **Figure 6A**. *EGFR* gene mutation (19del and L858R), *TP53* gene mutation, and *EGFR* amplification were the most common gene variations. Other concurrent gene mutations were also detected, including *BIM*, *CTNNB1*, and *PIK3CA* gene mutations. Six gene mutations in pretreatment samples, including *CDKN1B* amplification, *ERBB2* amplification, *ERBB2* mutation, *KMT2D* mutation, *PTEN* mutation, and *SETD2* mutation were significantly associated with PFS (**Supplementary Table 3**). The top 30 somatic mutations profile at PD point were listed in **Figure 6B**. The resistant mutation profile of the third-generation TKIs was shown in **Figure 6C**, in which *EGFR* C797S mutation was the most common resistant mutation (24%). *MET* CNV and *ERBB2* p.S22I and p.E992K mutations were also found in 3 and 5% patients, respectively. Moreover, 18 genes detected at PD were statistically significantly correlated with PFS (**Supplementary Table 4**), showing the complex mechanisms of drug resistance. These results suggest that dynamic cfDNA analysis is helpful in monitoring resistance to targeted therapy.

## Discussion

This study evaluated the role of dynamic cfDNA level based on a large-panel NGS with 425 genes in predicting outcomes and identifying possible resistant mutations in *EGFR* T790M-positive NSCLC patients who received the third-generation EGFR TKIs. Our results demonstrated that cfDNA clearance significantly correlated the longer PFS and OS of the third-generation EGFR TKIs. Moreover, cfDNA analysis depicted the resistant landscape during TKI treatment, providing guidance for successive treatment options.

Several studies have shown that cfDNA clearance during first-line EGFR TKI treatment predicts outcomes for first- and second-generation TKI therapy (22, 23). One of the studies showed that depletion of fDNA predicted outcome of patients with *EGFR* mutant NSCLC who received first-generation EGFR TKI using Cobas^®^ EGFR mutation test v2 (24). Song et al. reported that cfDNA clearance was associated with longer PFS in a real-world NSCLC cohort involving different kinds of treatment strategies using a 168-gene panel (25). But there was a lack of studies concerning the role of dynamic cfDNA level during the third-generation TKI treatment. A case report showed that clearance of fDNA-based ddPCR can predict the response to osimertinib (26). Ebert et al. also reported that clearing of all *EGFR* cfDNA after osimertinib treatment predicts the efficacy of Osimertinib with significant improvement in PFS, ORR, and DCR in 82 patients with NSCLC using Cobas^®^ EGFR mutation test v2 (27). Another study showed that cfDNA presence at 6 weeks correlated with short PFS and OS in a phase 1/2 study of osimertinib plus bevacizumab as first-line therapy for advanced EGFR-mutant NSCLC using the QX200 BioRad PCR system (28). Buder A et al. reported that cfDNA at initial and 8 weeks after osimertinib treatment in advanced EGFR-positive NSCLC was associated with shorter PFS and OS using droplet digital PCR (29). However, the limitations of the above studies were more likely due to the method of cfDNA testing, such as PCR, a non-quantitative analysis of cfDNA focusing on the limited specific mutation sites, a small sample size, or a smaller gene panel involving over 100 genes. Another important difference was that previous studies focus on cfDNA of *EGFR* genes, while our study analyzed the total cfDNA level detected by NGS. Our study demonstrated that clearing of cfDNA predicts the longer PFS and OS of the third-generation EGFR TKIs using a large gene NGS panel including 425 genes, and T790M level was not associated with outcomes. These results indicated that cfDNA was a positive predictor of prognosis of patients who received the third-generation TKIs.

Early clearance of plasma *EGFR* mutations (Ex19del, L858R and T790M) has been reported as a predictor of response to osimeritinb in both pretreated and treatment-naïve patients in different clinical trials (30–32). In this study, the correlation between *EGFR* T790M mutation and prognosis has been further discussed. In AURA3 trial, T790M-positive patients verified by tissue samples had a longer PFS than those verified in plasma (33), which indicated detectable plasma T790M status could be a reflection of tumor burden. The relative mutation purity of T790M (defined as the ratio of allelic frequency to maximum somatic allele frequency) was associated with significantly longer PFS of osimertinib treatment using small panel NGS (34). Bordi P et al. reported that patients with lower *EGFR* sensitive mutation ctDNA level (<2,200 copies/ml or allele frequency (AF) <6.1%) had a better PFS of osimertinib in plasma by ddPCR and Therascreen^®^ (10). In our study, in univariate analysis, pretreatment T790M level and level change during treatment course were associated with the PFS of the third-generation TKIs, but both were not confirmed in multivariate analysis which might be due to the confounding factors.

In clinical practices, the diagnosis of disease progression largely relied on radiological imaging. However, whether plasma cfDNA T790M can be employed as a valuable marker for changing the treatment strategy remains unknown. Prospective clinical trials on treatment strategy based on cfDNA level are highly expected. We observed that the re-detectable or re-elevated cfDNA level occurred 4.8 months earlier than progression on medical imaging, indicating molecular events develop earlier than clinical changing, which is accord with the previous studies focusing on cfDNA detection in early cancer relapse (35–37).

The cfDNA analysis based on NGS testing was also valuable in monitoring targeted drug resistance during rebiopsy. The resistant mechanism of osimertinib in pretreated and treatment-naïve patients has been shown to be different (38–40). In pretreated patients, *MET* amplification was observed in 19% of patients at disease progression and/or discontinuation with concurrent *EGFR* C797S (7%) and *EGFR* C797S+HER2 amplification (1%). In those samples with *MET* amplification, there was an almost equal split between samples that lost T790M (43%) *versus* those that retained T790M (57%) at disease progression and/or discontinuation. In treatment-naïve patients, the most frequently observed Osimertinib-resistant alterations were *MET* amplification (15%) and *EGFR* C797S (7%). Here in this study, the frequency of MET amplification was relatively low (3%), whereas the incidence of EGFR C797S mutation was higher than previously reported (24%). Meanwhile, cell cycle gene alterations such as *CDK6* CNV were presented at higher ratio compared to previous studies. Here *ABCB1* gene mutation which has been demonstrated as one of the resistant mechanisms of Osimertinib was also identified at a ratio of 5% (41, 42). These discrepancies in the detection of resistant mechanism could be due to the different panel using in each study as well as difference in the ethnic background of patients enrolled.

It is worth mentioning that several novel resistant gene alterations are firstly reported in our study, including *CDK6 CNV, GRIN2A, BRCA2, EGFR* D761N, *EGFR* Q791H, *EGFR* V843I, and *ERBB4* gene alteration. *CDK6* CNV has been reported to correlate with first-generation EGFR TKI resistance and shorten PFS to osimeritinib (43). The clinical impact of rare and compound mutations of *EGFR* including *EGFR* D761N, *EGFR* Q791H, *EGFR* V843I has been mentioned in EGFR TKI treatment, however, without conclusions thus far (44). *ERBB4* alterations have also been reported in NSCLC and found to be activated (45). Further studies are warranted to validate these potential resistant gene alterations. Besides, six gene alterations have been found at pretreatment and 18 gene alterations at PD point. The PFS of patients with these novel resistance gene alterations was shorter than that of patients without the gene alterations based on the small sample sizes, which needs validation in larger cohort and further studies.

Although plasma cfDNA-based re-biospy showed advantage in identifying off-target genetic resistance, histologic transformation which is a frequent early resistance mechanism to first-line osimertinib still might require tissue re-biopsy for further evaluation, especially for patients with co-mutation of *EGFR, TP53* and *RB1* (46). To overcome these emerging resistance mechanisms during osimeritinib treatment, combinational approaches have been proposed including CDK4/6 dual inhibitor plus osimertinib (47), MET-TKI plus osimertinib (48), checkpoint inhibitor plus EGFR TKI (49) in preclinical and clinical settings.

There are some limitations in our study. Firstly, not all of the patients had matched tissue and dynamic plasma samples resulting in partial data missing. The lack of matched tissue and dynamic plasma to compare the genetic background of multiple samples in the same patients partially invalidated the result obtained. Secondly, not all of the patients had the re-biopsy tumor tissue at resistant point of the third-generation EGFR TKIs. But censored data was allowed in terms of statistics. The over survival follow-up is on the way. Thirdly, the detailed function of these newly identified resistant gene alterations is unclear, which requires more functional studies.

Taken together, we observed the association of cfDNA clearance with the better PFS and OS in T790M-positive advanced NSCLC patients who received the third-generation EGFR TKIs through serial NGS testing. The dynamic plasma cfDNA level may be helpful for monitoring tumor response, predicting patient outcomes, and identifying resistance mechanisms during the third-generation EGFR TKI treatment in clinical practice. Prospective clinical trials of guiding the treatment strategy based on cfDNA level during targeted therapy are expected.

## Data Availability Statement

The data presented in the study are deposited in the Genome Sequence Archive for Human (GSA) repository, accession number HRA000725. The link is https://bigd.big.ac.cn/gsa-human/.

## Ethics Statement

The studies involving human participants were reviewed and approved by the ethics committee of Beijing Chest Hospital. The patients/participants provided their written informed consent to participate in this study.

## Author Contributions

JW, SZ, and LM designed the study and interpreted the results. LM, HL, DW, YH, MY, QZ, NQ, XZ, XL, HZ, YW, JL, and XY enrolled the patients and collected the clinical data. JW, LM, and HL wrote the manuscript. RY contributed to the manuscript revision–manuscript editing as well as data analysis during revision. All authors contributed to the article and approved the submitted version.

## Funding

This study was supported by the Capital Health Development Scientific Research Fund-the young talents program (2018-4-1043), Beijing Municipal Administration of Hospitals’ Youth program (QML20181602).

## Conflict of Interest

RY is an employee of Nanjing Geneseeq Technology Inc.

The remaining authors declare that the research was conducted in the absence of any commercial or financial relationships that could be construed as a potential conflict of interest.

## Supplementary Material

Supplementary Figure 1 Analysis of progression-free survival of the third-generation EGFR TKIs treatment among those with undetectable cfDNA level and those with detectable cfDNA within post-treatment of 1, 2, 3, or more than 3 months. **(A)** PFS stratified by different cfDNA level within 1 month after treatment. **(B)** PFS stratified by different cfDNA level within 2 months after treatment. **(C)** PFS stratified by different cfDNA level within 3 months after treatment. **(D)** PFS stratified by different cfDNA level more than 3 months after treatment.

Supplementary Figure 2 Correlation of cfDNA clearance duration with progression-free survival in patients treated with the third-generation EGFR TKIs.

Supplementary Figure 3 Analysis of progression-free survival of the third-generation EGFR TKIs treatment among those with undetectable T790M level and those with detectable T790M within post-treatment of 1, 2, 3, or more than 3 months. **(A)** PFS stratified by different T790M level within 1 month after treatment. **(B)** PFS stratified by different T790M level within 2 months after treatment. **(C)** PFS stratified by different T790M level within 3 months after treatment. **(D)** PFS stratified by different T790M level more than 3 months after treatment.

Supplementary Figure 4 Correlation of *EGFR* T790M clearance duration with progression-free survival in patients treated with the third-generation EGFR TKIs.

Supplementary Figure 5 Association of dynamic cfDNA level with imaging presentation of patients who developed resistance to the third-generation EGFR TKIs.
