# Supplementary figures and images for: Dynamic cfDNA Analysis by NGS in EGFR T790M-Positive Advanced NSCLC Patients Failed to the First-Generation EGFR-TKIs
Source: Front Oncol. 2021 Mar 25;11:643199. doi: 10.3389/fonc.2021.643199 (PMC8030263; doi:10.3389/fonc.2021.643199)

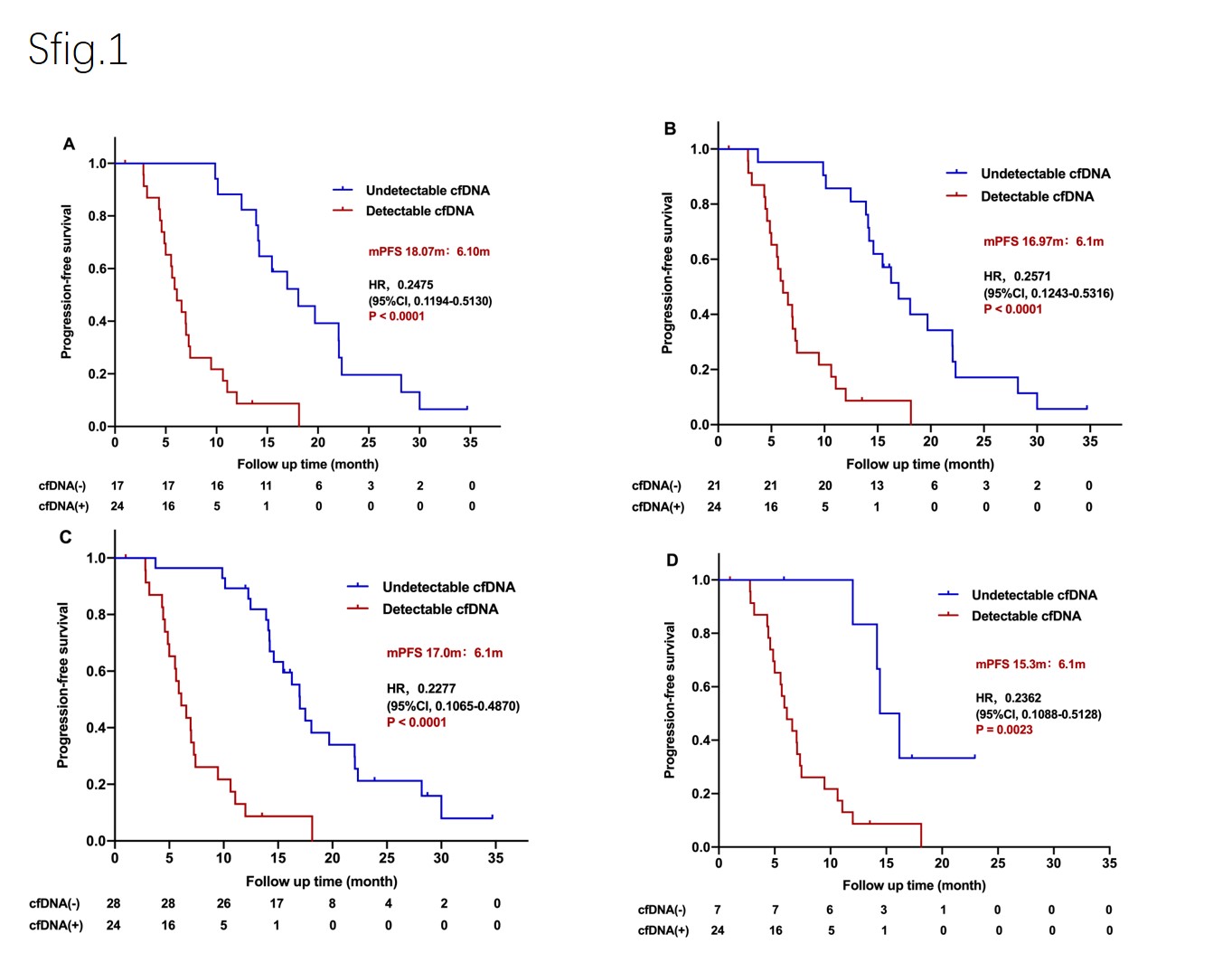

Supplement: Supplementary Figure 1 — Analysis of progression-free survival of the third-generation EGFR TKIs treatment among those with undetectable cfDNA level and those with detectable cfDNA within post-treatment of 1, 2, 3, or more than 3 months. (A) PFS stratified by different cfDNA level within 1 month after treatment. (B) PFS stratified by different cfDNA level within 2 months after treatment. (C) PFS stratified by different cfDNA level within 3 months after treatment. (D) PFS stratified by different cfDNA level more than 3 months after treatment. [file Image_1.jpeg]

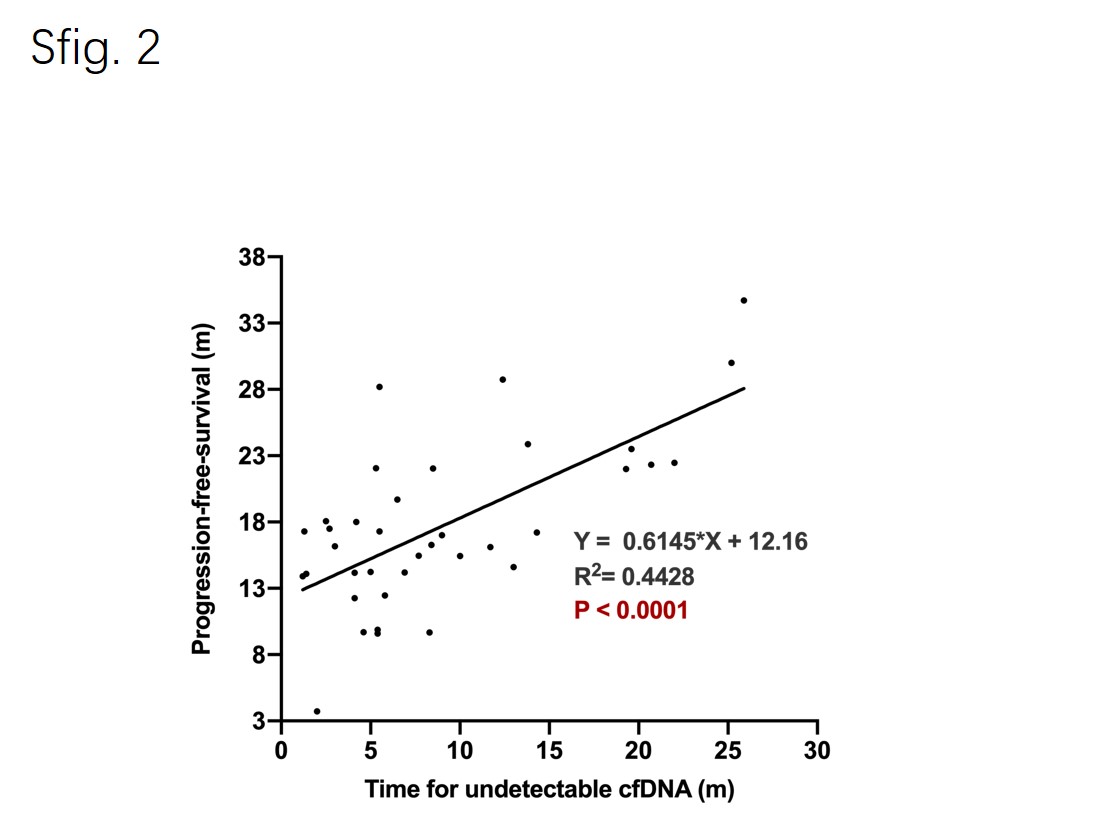

Supplement: Supplementary Figure 2 — Correlation of cfDNA clearance duration with progression-free survival in patients treated with the third-generation EGFR TKIs. [file Image_2.jpeg]

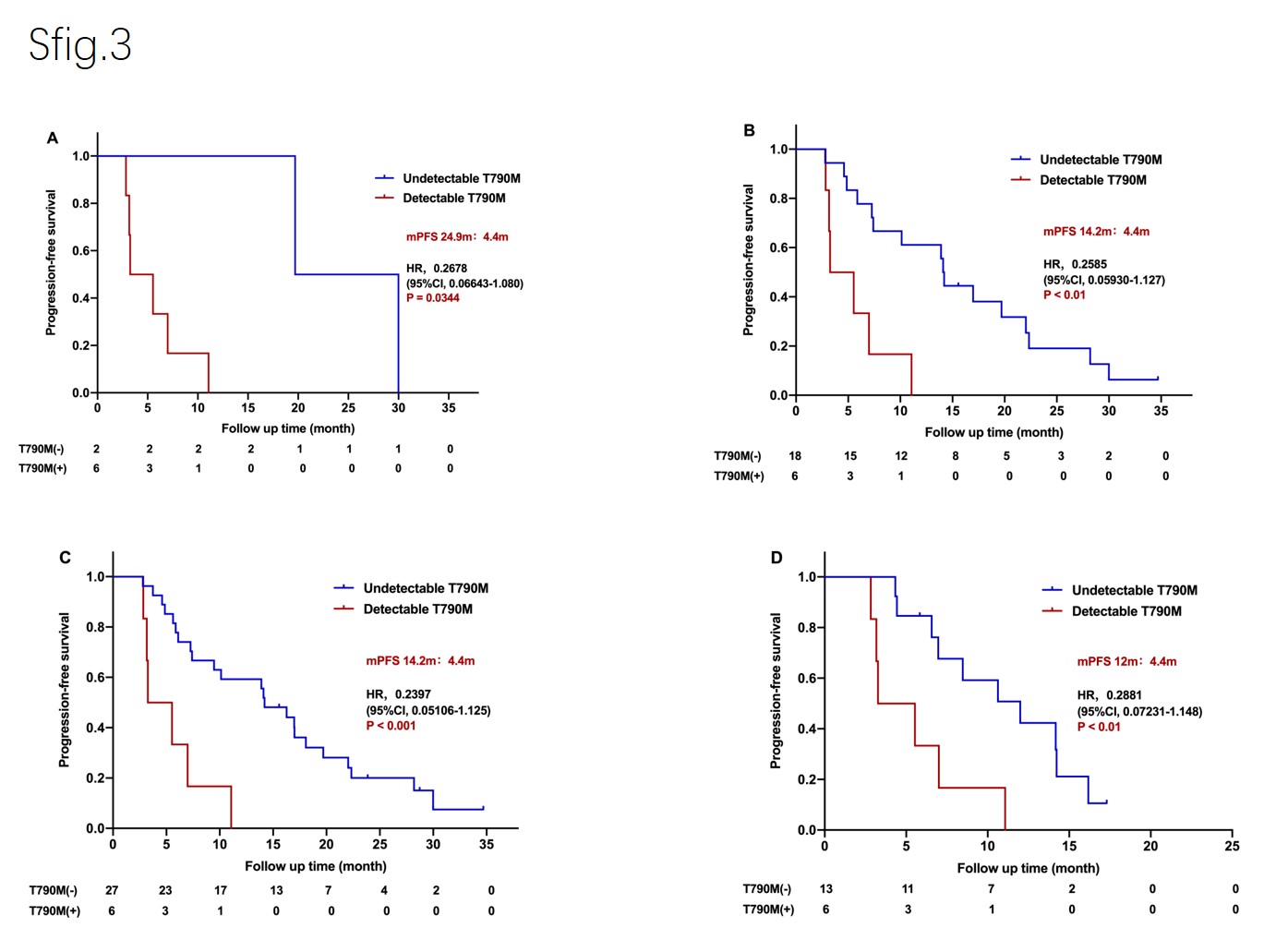

Supplement: Supplementary Figure 3 — Analysis of progression-free survival of the third-generation EGFR TKIs treatment among those with undetectable T790M level and those with detectable T790M within post-treatment of 1, 2, 3, or more than 3 months. (A) PFS stratified by different T790M level within 1 month after treatment. (B) PFS stratified by different T790M level within 2 months after treatment. (C) PFS stratified by different T790M level within 3 months after treatment. (D) PFS stratified by different T790M level more than 3 months after treatment. [file Image_3.jpeg]

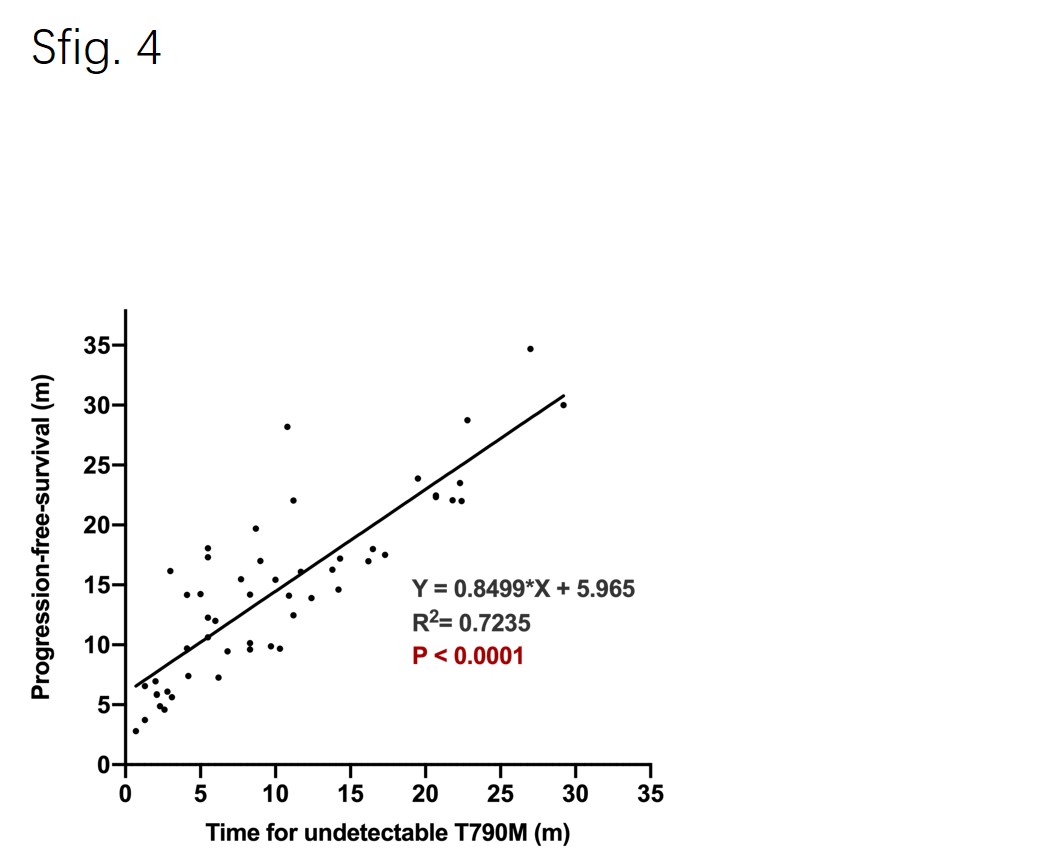

Supplement: Supplementary Figure 4 — Correlation of EGFR T790M clearance duration with progression-free survival in patients treated with the third-generation EGFR TKIs. [file Image_4.jpeg]

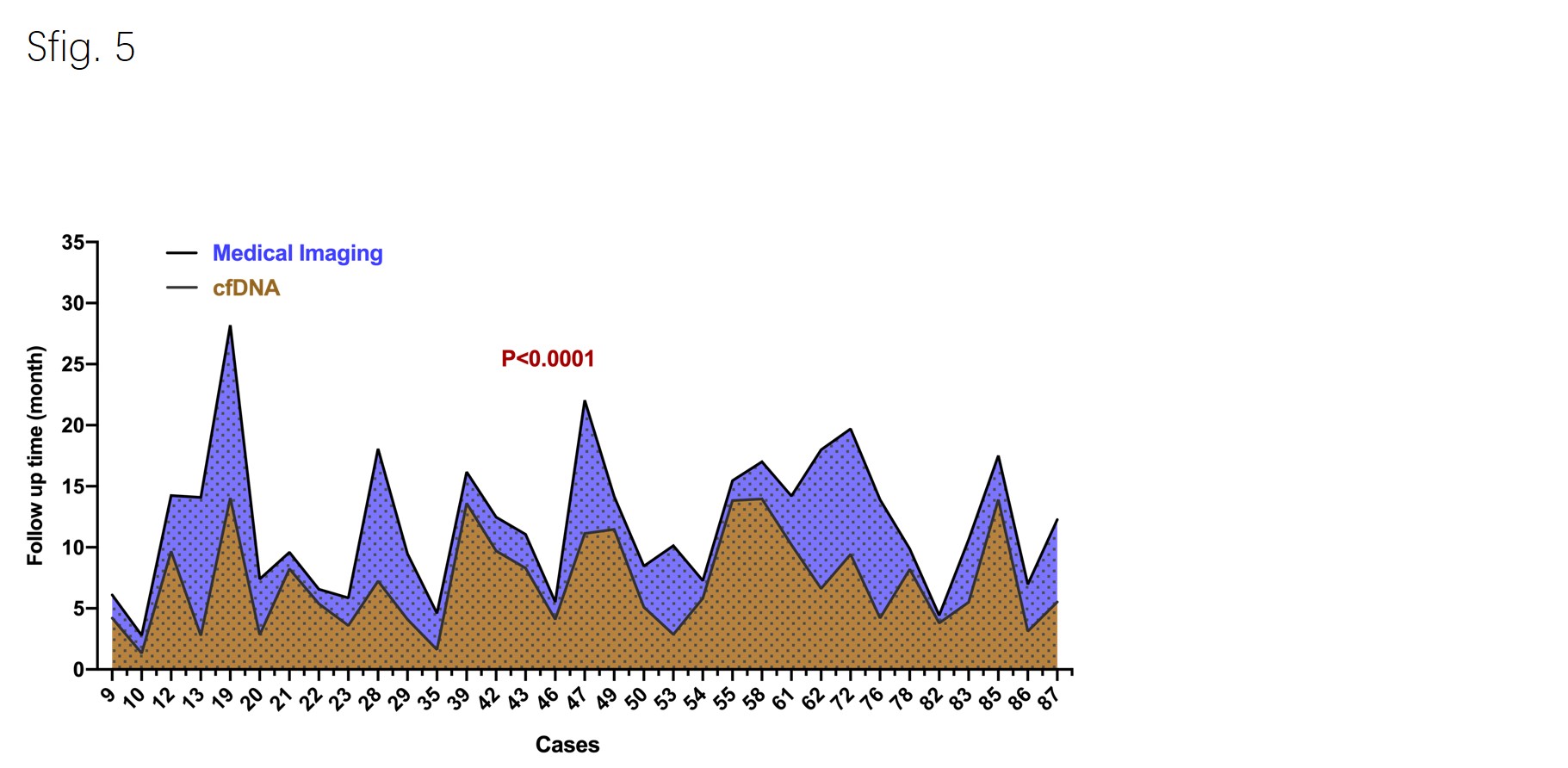

Supplement: Supplementary Figure 5 — Association of dynamic cfDNA level with imaging presentation of patients who developed resistance to the third-generation EGFR TKIs. [file Image_5.jpeg]
